# Shear Band-Induced Internal Surface Structures in a Vitreloy Bulk Metallic Glass Deformed by High-Pressure Torsion

**DOI:** 10.3390/ma18051096

**Published:** 2025-02-28

**Authors:** Zsolt Kovács, Talaye Arjmandabasi, Gábor Erdei, Erhard Schafler, Ádám Révész

**Affiliations:** 1 Department of Materials Physics, Institute of Physics, Eötvös Loránd University, Pázmány Péter St. 1/a, H-1117 Budapest, Hungary; kovacs.zsolt@ttk.elte.hu (Z.K.); t-arjmand@student.elte.hu (T.A.); 2 Department of Atomic Physics, Institute of Physics, Budapest University of Technology and Economics, H-1111 Budapest, Hungary; 3 Faculty of Physics, University of Vienna, Boltzmanngasse 5, A-1090 Vienna, Austria; erhard.schafler@univie.ac.at

**Keywords:** bulk metallic glass, severe plastic deformation, high-pressure torsion, optical profilometry, z-contrast surface imaging

## Abstract

In the present investigation, high stability Vitreloy Zr_44_Ti_11_Cu_10_Ni_10_Be_25_ bulk metallic glass has been subjected to severe shear deformation by high-pressure torsion for 0.1 revolutions under an applied pressure of 4 and 8 GPa. The fully glassy nature of the as-cast glass has been confirmed by X-ray powder diffraction and differential scanning calorimetry. Deformation-induced surface features on an internal plane of the deformed disk-shaped specimens were studied in detail at the macroscopic level by optical reconstruction method and at microscopic scales by white-light optical profilometry. Shear and compressive strain components were measured based on surface changes and it was determined that compressive strain gradient with 0.2–0.4 strain change builds up toward the disk edge, while only part of the nominal shear deformation occurs in the disk interior. The effect of strain localization in the Vitreloy bulk metallic glasses has been quantified by a surface distortion model based on simple shear. The model was then validated experimentally by the reconstructed z-profiles.

## 1. Introduction

Due to the lack of long-range atomic order, metallic glasses (MGs) possess exceptional mechanical properties, such as high strength, extreme elastic limit, excellent corrosion and wear resistance and have gained special interest in the last decades [1,2,3,4]. Nevertheless, the breakthrough of MGs as candidates for structural materials has not occurred yet due to their brittle behavior at low temperature and the lack of any detectable plasticity [5,6]. These special characteristics of MGs are directly related to their local atomic structure and the absence of crystalline defects [7]. Classically, MGs have been synthesized by rapid quenching from the molten state [8]. Above a critical cooling rate the disordered structure of the liquid is quenched-in at the glass transition temperature during the solidification that occurs without crystallization in the metastable supercooled liquid range. Recently, systems of specific composition have been developed that exhibit critical cooling rate in the range of 1–10 Kmin^−1^. These alloys can be cast into large volumes by surpassing crystallization during cooling [1,3]. All dimensions of these bulk metallic glasses (BMGs) can exceed several centimeters. Among the numerous multicomponent BMG classes, the Zr-based system with high glass forming ability (GFA) [9,10] has already been applied as structural components in the industrial sector, mainly due to its moderately expensive elemental components [3]. These BMGs also show good thermal stability [11,12] and excellent mechanical properties, including hardness, yield strength below their glass transition temperatures and creep in the supercooled liquid range [13,14,15].

Interestingly, BMGs may also possess inherent plasticity below the glass transition temperature under specific conditions, which can be manifested by mechanical constraints of different deformation geometry. Notable BMG plasticity can be reached in large sample volumes by severe plastic deformation (SPD) [16] during torsion [17] and bending [18]. Among other SPD methods, high-pressure torsion (HPT) was originally applied to synthesize porosity-free bulk ultrafine-grained materials, achieving an extremely large plastic deformation of up to γ ~ 100 [19,20]. In brief, during the HPT process a disk is placed between two hardened stainless-steel anvils and subjected to simultaneous hydrostatic pressure under several GPa and torsional straining for several rotations [21]. In recent years HPT has successively served as an ideal method by which to induce large plastic strain in different BMG systems [22,23], including Zr-based alloys [12,24,25,26,27,28].

Low-temperature plasticity and brittle failure in BMGs occur mostly by the operation of shear bands [1,29]. These shear bands are extremely thin, with nanometer-wide planes [30] exhibiting localized plastic deformation. The formation procedures of shear bands are not fully known, but shear-induced softening by the formation of less strong atomic environments has an important role in severe strain localization [31,32]. Nevertheless, a minor strain hardening effect can also be observed in some BMG systems [33]. Macroscopically, shear displacement (offset) can be observed between the two sides of a shear band. Spatial variation of this offset indicates the strain and stress fields, which have an important role in the evolution of the shear bands [34]. The inherent softening in shear banding of BMGs can also induce inhomogeneities at macroscopic length scales. Variation of shear strain along the HPT axis and deviation from nominal deformation were observed in HPT-deformed disks [35], in contrast with the more homogeneous plastic deformation observed in fine-grained crystalline material [36].

In the present study, optical profilometry techniques were applied on high stability Vitreloy Zr_44_Ti_11_Cu_10_Ni_10_Be_25_ bulk metallic glass disks deformed by high-pressure torsion. Deformation-induced surface features were investigated on internal sections, both at the macroscopic and microscopic scales, via z-imaging reconstruction method and white-light optical profilometry, respectively. To quantify the effect of strain localization, a surface distortion model based on simple shear has been developed and compared with the experimentally reconstructed z-profiles. The novelty of the present study is the analytical approach with which the deformation field of the HPT deformation is characterized. Based on this approach, not only are the effects of the shear and compression components separated in the HPT-deformed Vitreloy BMG, but local variations along shear bands are quantitatively characterized.

## 2. Materials and Methods

### 2.1. Sample Preparation

**Figure 1:**
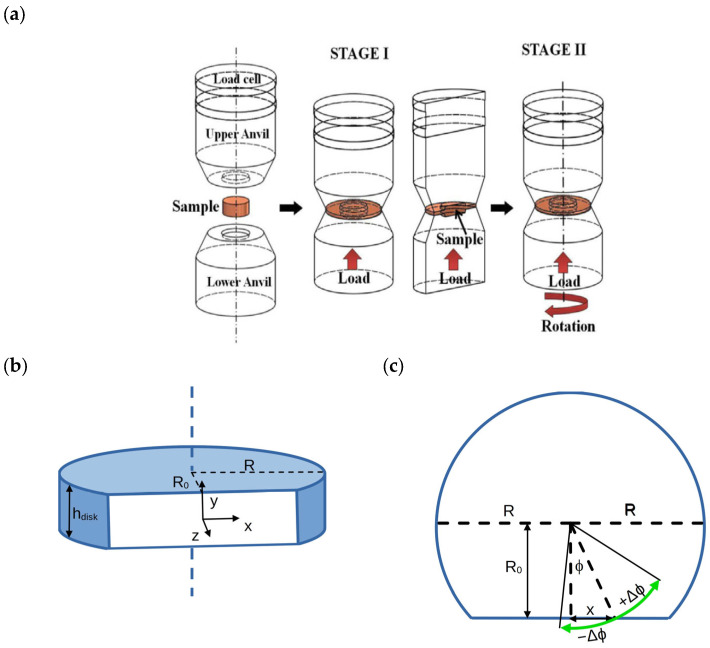
(**a**) Schematic representation of the HPT apparatus with the uniaxial compression (I) and simultaneous compression and torsion (II) stages [37]. (**b**) Schematic drawing of the major sample part with the sample parameters and the surface which was opened up after HPT deformation. (**c**) Plane view of the major part of the sample disk with the *ϕ*(*y*,*z*) local rotation angle characterizing the surface distortion after HPT deformation.

Commercial Vitreloy Zr_44_Ti_11_Cu_10_Ni_10_Be_25_ (Vit 1bTM; Liquidmetal Technologies Inc., Rancho Santa Margarita, CA, USA) bulk metallic glass was subject to severe plastic deformation by high-pressure torsion under air at room temperature. A schematic illustration of the HPT apparatus is visualized in Figure 1a. Disks of the as-cast BMG with a radius and height of *R* = 3 mm and *h_disk_* = 0.8 mm, respectively, were machined by spark erosion. Then, these disks were cut into major and a minor parts asymmetrically parallel to the axis of the disk in a radial distance of approximately *R*_0_ = 1–1.4 mm along a secant (Figure 1b). The cut surfaces were mirror polished using 50 nm Al_2_O_3_ powder. The reconstructed disks were placed between two stainless-steel anvils of an HPT apparatus. Torsion experiments were conducted under applied pressures of *p* = 4 GPa and 8 GPa, with an angular velocity of 0.2 rev/min up to *N* = 0.1 turns under constrained geometry.

In general, the accumulated shear strain (*γ*) for torsion deformation at a radius *r* can be given by

(1) $$\gamma \left(r\right)=\frac{\theta r}{h_{disk}}=\frac{2\pi Nr}{h_{disk}},$$

where *θ* is the rotation angle (presently *θ* = 0.628 rad) between the top and bottom of the disk. Figure 1c shows the effect of the surface rotation (green arrows) schematically for the major disk part in which a rotation of θ=2Δϕ is achieved between −Δϕ and Δϕ. To demonstrate the effect of strain softening in BMGs, two samples were deformed under 4 GPa and two samples under 8 GPa hydrostatic strain in the present experiment. For each sample, the *γ*_0_ nominal strain was also calculated as *γ*(*r* = *R*) based on the original machined parameters of the disks. Notations and characteristic parameters of the different sample disks are listed below in Table 1. Based on former experience [35], HPT of some BMGs show notable scatter in the experimental plastic strain in comparison with the respective nominal strain; therefore, in this case, two disks, i.e., samples HPT4A and HPT4B, were produced with the same characteristic parameters.

### 2.2. Characterization of the As-Cast Glass

The overall structure of the Vitreloy BMG was examined by X-ray powder diffraction (XRD). The measurements were carried out on a Rigaku SmartLab (Rigaku, Tokyo, Japan) diffractometer applying Cu-Kα radiation in *θ*–2*θ* geometry in a range from 2*θ* = 25° to 100°, with a step size of 0.01°.

The crystallization performance of the as-cast glass was investigated by a Perkin Elmer power-compensated differential scanning calorimeter (DSC) (Perkin Elmer, Shelton, CT, USA) in a linear heating ramp carried out at 20 Kmin^−1^. The enthalpy released during crystallization was obtained as the area of the exothermic peaks after baseline correction. The measurement was performed under a protective Ar atmosphere.

### 2.3. Surface Characterization

**Figure 2:**
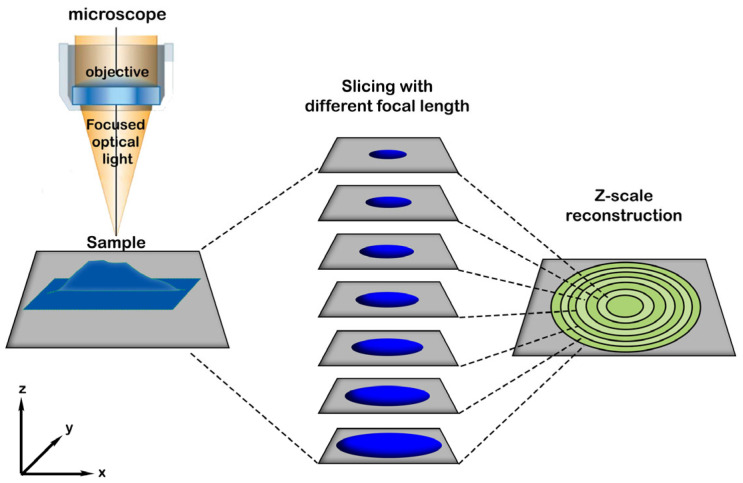
Schematic representation of the optical imaging and z-image reconstruction in slicing mode.

A series of optical images was captured with 5× magnification at 64 equidistant focal lengths for each sample surface with a Zeiss Axio Imager M2 optical microscope (Zeiss, Oberkochen, Germany) in slicing mode (Figure 2). From this image series, an optical image of the sample surface was reconstructed based on the details which were in focus for each segment of the images. The in-focus image segments also provided the height (*z*) of the image feature in the segments. From this information the surface of the sample was reconstructed, which is referred hereafter as the *z*-image. To produce full disk images from higher magnification localized images (both reconstructed optical and *z*-images) the free GIMP software (version number: 2.10.30) [38] was used.

Fine details of the surface topography were measured by a Bruker Contour GT-K0X (Bruker, Billerica, MA, USA) white-light optical profilometer, which is practically an interference microscope. The equipment implements short coherence length interferometry with a Mireau-type microscope objective, which creates a reference flat that is virtually inside the test object. An interferogram is formed between the reference surface and the object surface. Due to the short coherence length of the applied illumination, only a few fringes are visible around points where the optical path length between the virtual reference flat and the test object is zero. To measure a surface area of about 160 μm × 120 μm, the microscope’s 50× objective, together with the virtual reference, is scanned in the *z*-direction, i.e., orthogonally to the sample’s surface, and at each position a 2D camera image is captured from the surface. After the scan is completed, interferograms are obtained from the image series in each pixel and the interferograms are evaluated in an automated procedure to determine the z coordinate of the surface at that particular pixel. In our tests, the uncertainty of the determined z coordinates was less than 10 nm and accuracy was about 0.8%. For these measurements with a 50× objective, lateral resolution of the topography images was about 500 nm, while the spatial sampling pitch in the object plane was cca *q* = 130 nm. Quantitative analysis of the 3D surface topography was performed using the free Gwyddion software (version number: 2.67) [39].

## 3. Results

### 3.1. Structural and Thermal Analysis of the As-Cast BMG

**Figure 3:**
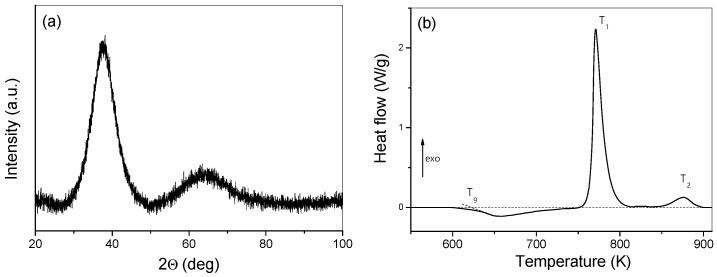
(**a**) Powder X-ray diffractogram and (**b**) linear heating calorimetric measurement of the as-cast Vitreloy BMG. Dotted line represents the calorimetric baseline.

The XRD pattern of the as-cast Vitreloy glass is characterized by two broad diffraction halos, together with the lack of any crystalline Bragg reflection, confirming that the material is X-ray amorphous (Figure 3a).

The linear heating DSC thermogram reveals the endothermic glass transition (*T_g_* = 620 K) and a two-step exothermic crystallization feature characterized by *T*_1_ = 771 K and *T*_2_ = 876 K transformation temperatures (Figure 3b). The corresponding supercooled liquid region ∆*T_x_* ~ 130 K (∆*T_x_* = *T_1_*_, *onset*_ − *T_g_*) refers to an extremely high glass forming ability (GFA), one of the largest among all metallic glass systems. Such values indicate that the undercooled liquid has a very strong resistance against devitrification and further crystallization either by thermal activation or by plastic deformation [12,24]. In addition, the total crystallization enthalpy release (Δ*H* = 108 Jg^−1^) confirms that the Vitreloy BMG is highly metastable.

### 3.2. Optical Imaging of the Internal Surface of HPT Disks

**Figure 4:**
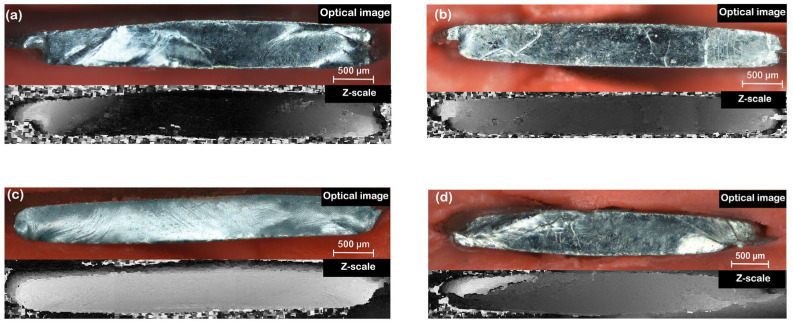
Optical images (upper images) and the corresponding z-scale (lower images) of the (**a**) HPT8A, (**b**) HPT8B, (**c**) HPT4A and (**d**) HPT4B samples.

BMG disks were unloaded after plastic deformation of the HPT process and the two parts were separated from each other. Internal surfaces of the larger pieces were investigated by optical methods. These surfaces are curved due to the plastic deformation, which can nominally reach *γ* = *γ*_0_ at the perimeter of the disk. In the case of curved surfaces, an optical reconstruction method was used (detailed in Section 2.3) to remove the focusing problems and obtain an in-focus image of the curved surface. Figure 4 shows the reconstructed optical images of the internal surfaces of the HPT disks along with the z-scale surface height images. Meanwhile, the total grayscale height range of 0–255 (from black to white) corresponds to height differences of 610, 498, 962 and 1346 μm in the z-scale images for the HPT8A, HPT8B, HPT4A and HPT4B samples, respectively.

### 3.3. Optical Profilometry of the Internal Surface of HPT Disks

**Figure 5:**
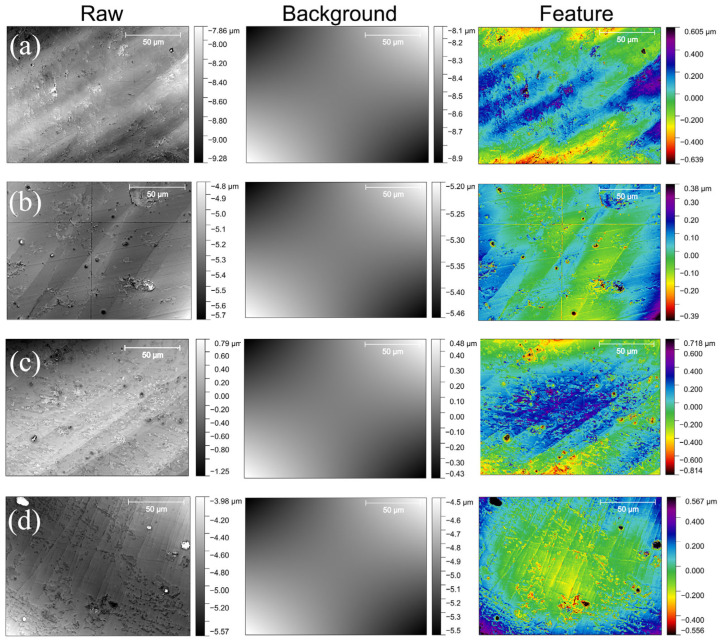
Optical profilometry images of the (**a**) HPT8A, (**b**) HPT8B (**c**) HPT4A and (**d**) HPT4B samples. For each sample a raw optical profilometry image after removal of the average plane (raw), a fitted bilinear background (background) and a colored feature image after the removal of the bilinear background (feature) is shown.

Local fluctuations of the surface height can be analyzed based on height maps produced by optical profilometry. These height maps, after the removal of a fit plain background, are shown as raw images in Figure 5. The optical profilometry images were obtained at the center of the internal surface of the HPT disks for the (a) HPT8A, (b) HPT8B (c) HPT4A and (d) HPT4B samples. Plain regions with sharp offsets and slight undulations can be observed through the imaged sample areas in all images. The appearance of this surface roughness indicates the formation of shear bands in these plastically deformed samples. Additionally, surface curvature due to the HPT deformation along the whole imaged area can also be observed in these raw images. To separate the surface curvature from the finer surface features the height map of each raw image was split into a background and a feature image as detailed in the discussion section below.

## 4. Discussion

In ideal plastic shearing, the initial plain surface should change in an HPT disk, in agreement with Equation (1). Therefore plastic deformation produces a specifically curved surface in each sample. To reveal differences between the ideal plastic shearing and the plastic shear deformation that appears in BMG disks during HPT experiments, the optically determined surface shapes and the shape determined from the ideal plastic shearing are compared in the following.

### 4.1. Simple Shear

Initial plain surface at R_0_ distance from the center of the disk axis can be described as *z*(*x*,*y*) = 0 in the coordinate system depicted in Figure 1b, where each position is represented by (*x*,*y*) coordinates along the internal surface of the major HPT disk part. These points are positioned at $r=\sqrt{R_{0}^{2}+x^{2}}$ distance from the axis of the disk and can be characterized by a $\phi =asin\left(x/\sqrt{R_{0}^{2}+x^{2}}\right)$ polar angle, which is measured from the normal vector of the surface plane. Surface displacements in ideal simple plastic shearing can be described in this polar coordinate system as $\Delta \phi \left(x,y\right)=\theta \;y/h$. Therefore, no y displacement (Δy=0) can be anticipated due to the shearing of the HPT deformation, while other surface displacement can be calculated as $\Delta x=rsin\left(\phi +\Delta \phi \right)-rsin\left(\phi \right)$ and $\Delta z=rcos\left(\phi +\Delta \phi \right)-rcos\left(\phi \right)$ in the Cartesian coordinate system of the initial plane surface. As Δϕ<θ/2=0.314rad, $sin\left(\Delta \phi \right)$ and $cos\left(\Delta \phi \right)$ can be approximated quite well in this range with linear and constant terms, respectively, the small x and z displacements of an (x,y) position at the internal surface of the HPT disk can be simplified as

(2) $$\Delta x\approx r\left[\frac{R_{0}}{\sqrt{R_{0}^{2}+x^{2}}}\frac{\theta y}{h}\right]=\frac{\theta R_{0}}{h}y=\frac{\gamma_{0}R_{0}}{R}y,$$

(3) $$\Delta z\approx r\left[\frac{-x}{\sqrt{R_{0}^{2}+x^{2}}}\frac{\theta y}{h}\right]=-\frac{\theta }{h}xy=-\frac{\gamma_{0}}{R}xy.$$

Equation (2), accounts for a homogeneous shear distortion along the cut surface plane, irrespective of the position, while Equation (3) predicts a bilinear curved surface shape with displacements in the z direction. Therefore, overall shear deformation of HPT disks can be simply determined experimentally from the surface curvature of the deformed HPT disk parts by applying a polynomial fit with bilinear coefficients in both the x and y coordinates, $\Delta z\approx P\left(x,y\right)=\sum_{i=0}^{1}\sum_{j=0}^{1}a_{ij}x^{i}y^{j}$, where $a_{ij}$ is constant. Selection of the exact zero position for the coordinate system produces only changes in the constant and linear parameters of P(x,y) and has no effect on the $a_{11}$ bilinear coefficient. Therefore fitting of P(x,y) to an arbitrary selected area provides an estimation on the average shear deformation of a specific area as $\gamma \left(x,y\right)=Ra_{11}\left(x,y\right)$ around the (x,y) position.

**Figure 6:**
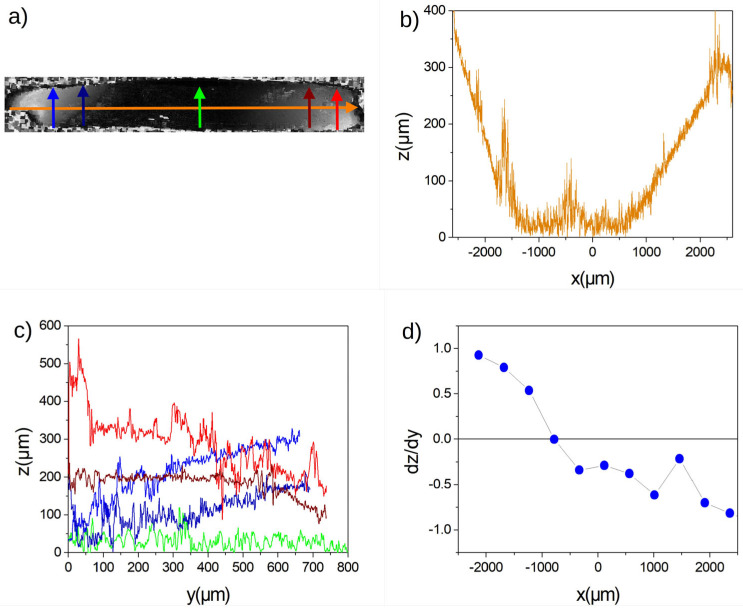
Profile analysis of HPT8A *z*-image. (**a**) The z-image, with color arrows indicating the specific trajectories for the profile analysis. (**b**) The *z* profile in the disk plane corresponding to the orange trajectory. (**c**) The *z* profiles parallel to the torsion axis measured at different distances from the center. Profiles match in color with the arrows in the *z* -image. (**d**) *dz*/*dy* profile slopes as a function of *x* position.

To validate Equation (3), specific z profiles from the HPT8A sample are analyzed in Figure 6. Figure 6a shows the z-image of HPT8A with color arrows indicating the specific trajectories for the profile analysis. According to Equation (3), the $\Delta z\left(y=0\right)$ surface displacement should be equal to zero for z profiles that lie in the symmetrical plane of the HPT disk. However, as one can notice from Figure 6b, $\Delta z\left(y=0\right)$ is valid only approximately in the vicinity of the center of the z-image indicated by the orange arrow in Figure 6a. Deviations from the zero background are related to the compression of the HPT disk, which will be discussed in Section 4.2. Figure 6c shows z profiles parallel to the torsion axis measured at different distances from the center (profiles match in color with the arrows in Figure 6a). These profiles exhibit positive or negative average slopes depending on the sign of the *x* position, as can be anticipated from Equation (3). As a function of the x position, the *dz*/*dy* profile slopes follow approximately a linear relationship, as depicted in Figure 6d. The slope of this *dz*/*dy* vs. x plot directly provides the bilinear parameter as $a_{11}=0.35\;{mm}^{-1}$, corresponding to an average value of $\gamma_{0}=1.05$. The difference between the nominal strain (see Table 1) and the measured $\gamma_{0}$ indicates that only 44% of the nominal strain occurs inside the HPT disk, while the majority of the shear deformation is generated within a thin surface layer at the contact of the anvil and the BMG disk [35].

### 4.2. Compression

**Figure 7:**
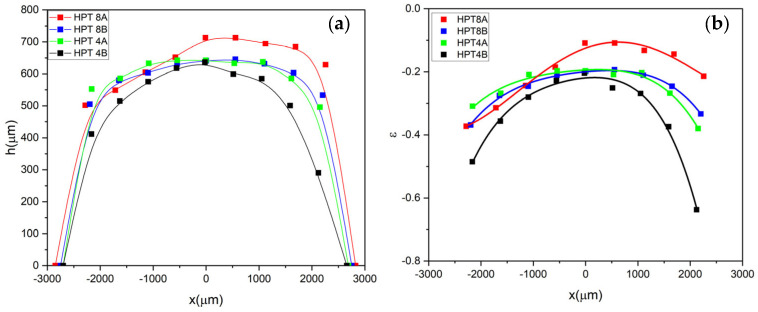
(**a**) Height profiles and (**b**) the calculated axial compressive strain distribution of the different HPT samples.

As a side effect in HPT deformation, axial compression towards the perimeter of the HPT disk produces a shape change when compared with the initial perfect disk geometry (see the elongated disks shapes in Figure 4). This shape change is necessary to reach a hydrostatic stress state by the externally applied uniaxial compression (see stage I in Figure 1a). Shape changes in the present HPT experiments are characterized quantitatively by the varying height profiles in Figure 7a.

Presuming that the cylindrical symmetry of the disks are preserved after the HPT deformation, $\Delta z=\left(r-r\varepsilon \left(r\right)/2\right)cos\left(\phi \right)-rcos\left(\phi \right)$ surface displacement can be predicted, where $\varepsilon \left(r\right)$ is the distribution of the axial (HPT axis) compressive strain along the disk radius. Taking into account the geometry constraints between r,ϕ,x and *R*_0_, the shape change in the y direction shifts the z position of the surface,

(4) $$\Delta z\approx -r\frac{\varepsilon \left(x\right)}{2}\left[\frac{R_{0}}{\sqrt{R_{0}^{2}+x^{2}}}\right]=-\frac{R_{0}}{2}\varepsilon \left(x\right),$$

where the strain distribution was calculated from the surface profiles as $\varepsilon \left(x\right)=\left(h\left(x\right)-h_{0}\right)/h_{0}$. The calculated strain distributions are shown in Figure 7b for all of the samples. As seen from Equation (4), if cylindrical symmetry is supposed in the deformation of the HPT disk, this Δz contribution should contain only even exponents in x and should produce surface height changes which are independent from the y coordinate. Thus, the effect of compression is best visible in the disk plane of the z-image, where shear effects on Δz can be neglected (see Figure 6b). As the anvils can displace their axes slightly during the extreme loads of the HPT process during either the compression phase of stage I or the torsion phase of stage II (see Figure 1a), the cylindrical symmetry of the disk is not strictly preserved during the HPT deformation. To quantify the shape of the different HPT samples and take also into account this asymmetry effect, the h(x) profiles were fit by a fourth order polynomial of the x coordinate, $h\left(x\right)=h_{0}+h_{1}x+h_{2}x^{2}+h_{3}x^{3}+h_{4}x^{4}$. The results of these fits are shown on the proportional strain distribution plot in Figure 7b. The fitted shape parameters are listed in Table 2. Based on the compression strain curves in Figure 7b, a deformation gradient builds up towards the disk edges and an approximate 0.2–0.4 strain change is visible in the outer part of the of HPT disks.

The spatial variation of compression and shear strain components raise the question about whether plastic strain pattern correlates with microstructural changes of the BMG alloy. These plastic deformation-induced structural changes are typically small but can be experimentally measured with high stability, position-sensitive methods as has been presenter prior [12].

### 4.3. Local Characterization of the Deformation

Optical profilometry images provide a high z resolution map of a local central region of the internal surface on the order of 100 μm. Due to the small error of the optical profilometry measurements, quantitative characterization of local surface profiles of the disks can also be performed based on Equations (2)–(4). The bilinear surface fits based on Equation (3) are shown in the background column in Figure 5. The corresponding a_11_ values are 0.078, 0.026, 0.052 and 0.102 mm^−^^1^ for the HPT8A, HPT8B, HPT4A and HPT4B samples, respectively. These values can deviate significantly from the global a_11_ parameters, because shear banding can induce strong local Δz offsets at specific regions of the samples. As seen in the raw profilometry images and also on the feature images which were obtained after the removal of the fit bilinear background (Figure 5), the samples do indeed have significant roughness at the (sub)micrometer scale. This surface roughness is the consequence of the spatially strongly varying plastic deformation component produced by shear banding in the BMGs [40]. Further analysis of this fine plastic deformation field is also possible based on Equations (3) and (4). To overcome the problem of producing area fits at various length scales to the strongly undulating surfaces, a local convolution method on the $\Delta z_{k,l}=\Delta z\left(x_{k},y_{l}\right)$ discrete surface maps was developed,

(5) $$D_{k,l}=\sum_{m=-1}^{1}\sum_{n=-1}^{1}C_{m,n}\Delta z_{k+m,l+n},$$

where $D_{k,l}=D\left(x_{k},y_{l}\right)$ is the calculated deformation map and $C_{m,n}$ is the convolution matrix:

(6) $$C_{m,n}=\left(\begin{array}{ccc} -1 & 0 & 1\\ 0 & 0 & 0\\ 1 & 0 & -1 \end{array}\right),$$

and the pixel size of the discrete maps are *q* = 200 nm, i.e., $x_{k+1}=x_{k}+q$ and $y_{l+1}=y_{l}+q$, with the exception of the coordinates of the neighboring data points. By calculating the D map for the special Δz surface shape obtained for simple shear based on Equation (3), one can obtain the following:

(7) $$D=a_{11}\left[\left(x+q\right)\left(y+q\right)+\left(x-q\right)\left(y-q\right)-\left(x-q\right)\left(y+q\right)-\left(x+q\right)\left(y-q\right)\right].$$

Thereafter, a position-independent constant D deformation map can be obtained as

(8) $$D=4a_{11}q^{2}.$$

Similarly, contribution of the Δz surface shape from axial compression (Equation (4)) leads to =0, because Δz depends only on the x coordinate and is independent from the y coordinate. Therefore, the calculated D map should be equal in spatial average $D=4a_{11}q^{2}$, where the $a_{11}$ parameter is determined from the global surface fit of Equation (3).

**Figure 8:**
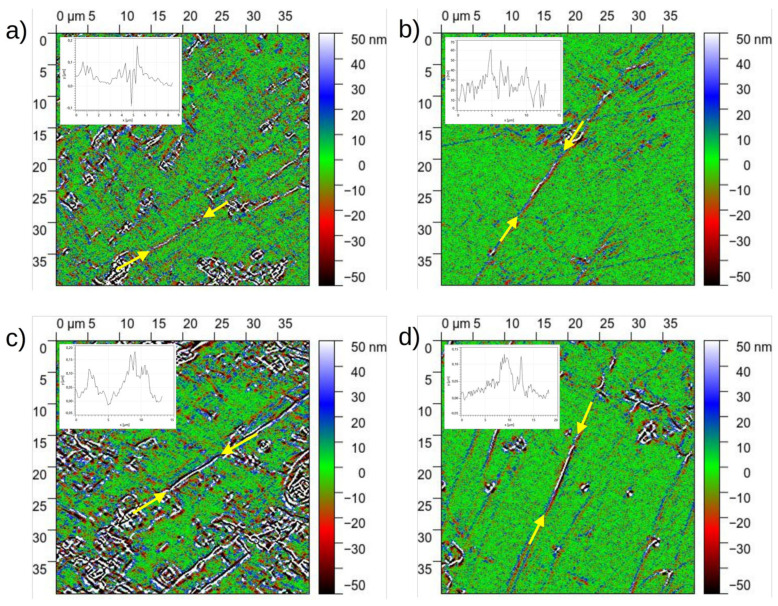
Selected area of D maps corresponding to (**a**) HPT8A, (**b**) HPT8B, (**c**) HPT4A and (**d**) HPT4B. The inset shows D profiles along the indicated shear bands.

Shear bands in BMGs are typically few nm wide regions in which plastic deformation is mainly localized [30,40,41]. As seen in Figure 8, the D maps show strong contrast (color different from green) in these straight regions. As the thickness of shear bands is smaller than the q pixel size, the D value map shows a negative (red) and a positive (blue) stripe with equal amplitude around the shear band along its two sides. The amplitude is proportional to the local Δz offset of the shear band, which can vary strongly along the shear band, as shown by the D profile in the inset measured along the indicated main shear bands. The strongly varying shear offset along a single shear band indicates that shear bands form in a stochastic process. It is noted that shear band patters tend to exhibit different features towards the periphery of a BMG HPT disk; however, detailed analysis of these features requires additional SEM and profilometry experiments and is therefore outside the scope of the present manuscript.

## 5. Conclusions

High-stability Vitreloy Zr_44_Ti_11_Cu_10_Ni_10_Be_25_ bulk metallic glass disks were deformed plastically by high-pressure torsion at two different hydrostatic pressures. HPT disks were composed of two parts and the internal surfaces of the deformed disks were characterized globally by slice reconstruction via optical microscope and locally via optical profilometry. The surface shape and curvature were determined quantitatively and compared with a model describing the surface features based on simple shear and compression. Local characterization of the surface profiles was carried out by a convolutional analysis that provided information on the local shear strain. The measured shear and compressive strain values show that only part (e.g., 44% in HPT8A) of the nominal *γ*_0_ = 2.36 shear strain is realized in the interior of the HPT samples, while a gradient in the compressive strain builds up towards the disk edges with 0.2–0.4 strain change in the outer part of the of HPT disks. The same analysis led to a simple tool which identifies shear band trajectories and which allows one to measure the out-of-plane shear offset along the shear band trajectories. Varying shear offset along a single shear band indicates the presence of stochastic process during shear band formation.

## Figures and Tables

**Table 1:** Characteristic parameters of the different sample disks.

Sample	Pressure, *p* (GPa)	Revolution, *θ* (rad)	Disk Height, *h_disk_* (mm)	Nominal Strain, *γ*_0_	Secant Distance *R*_0_ (mm)
HPT8A	8	0.1	0.8	2.36	1
HPT8B	8	0.1	0.8	2.36	1.4
HPT4A	4	0.1	0.8	2.36	1
HPT4B	4	0.1	0.8	2.36	1

**Table 2:** Fitted deformation shape parameters of the different sample disks.

Sample	*h*_0_ (mm)	*h*_1_ (-)	*h*_2_ (mm^−1^)	*h*_3_ (mm^−2^)	*h*_4_ (mm^−3^)
HPT8A	0.693	0.048	−0.036	−0.004	0.002
HPT8B	0.634	0.012	−0.011	−0.001	−0.003
HPT4A	0.645	0.007	−0.012	−0.004	−0.003
HPT4B	0.624	0.011	−0.030	−0.009	−0.006

## Data Availability

The original contributions presented in the study are included in the article, further inquiries can be directed to the corresponding authors.
